# Exploratory clinical trial to evaluate the efficacy and safety of transdermal electrical stimulation in patients with central retinal artery occlusion

**DOI:** 10.1371/journal.pone.0282003

**Published:** 2023-02-24

**Authors:** Gen Miura, Tadami Fujiwara, Takayuki Iwase, Yoshihito Ozawa, Yuki Shiko, Yohei Kawasaki, Tomohiro Nizawa, Tomoaki Tatsumi, Takayuki Baba, Takuji Kurimoto, Sotaro Mori, Makoto Nakamura, Hideki Hanaoka, Shuichi Yamamoto

**Affiliations:** 1 Department of Ophthalmology and Visual Science, Chiba University Graduate School of Medicine, Chiba, Japan; 2 Clinical Research Centre, Chiba University Hospital, Chiba, Japan; 3 Department of Surgery, Division of Ophthalmology, Kobe University Graduate School of Medicine, Kobe, Japan; Tsukazaki Hospital, JAPAN

## Abstract

**Purpose:**

To evaluate the efficacy and safety of transdermal electrical stimulation (TdES) using skin electrodes in patients with central retinal artery occlusion (CRAO).

**Methods:**

Five eyes of five patients with CRAO underwent TdES (10-ms biphasic pulses, 20 Hz, 30 min) six times at 2-week intervals. Only the affected eye was stimulated with 1.0-mA pulses in all patients. The primary endpoint was the best-corrected logMAR visual acuity. The secondary endpoints were changes in the best-corrected logMAR visual acuity, Early Treatment of Diabetic Retinopathy Study (ETDRS) visual acuity, mean deviation of the Humphrey field analyzer (HFA) 10–2, and HFA Esterman test score. We also evaluated its safety.

**Results:**

The logMAR visual acuity at 12 weeks was improved by 0.1 or more in two patients and was maintained in two patients compared to the baseline. No obvious changes in the mean logMAR visual acuity, ETDRS visual acuity, mean deviation, and HFA Esterman score were observed at 12 weeks compared to the baseline. All five enrolled patients completed the study according to the protocol. No treatment-related adverse events were observed during this study.

**Conclusion:**

In this study, logMAR visual acuity was slightly improved in two patients, confirming the safety of TdES. Since CRAO has no established treatment method, further research into the effects of TdES treatment in CRAO patients may be beneficial.

## Introduction

Electrical stimulation therapy has been reported to improve visual function in patients with retinitis pigmentosa (RP) [1], age-related dry macular degeneration [2], glaucoma [3], and optic neuropathy [4]. Some reviews have provided evidence of electrical stimulation of the visual system and insight into electrical stimulation as a therapy for various ocular diseases [5,6].

Previous studies have shown that the improvement of visual function by ocular electrical stimulation is related to the improvement of survival rate and functional activation of retinal ganglion cells by the activation of insulin-like growth factor-1 [7], brain derived neurotrophic factor [8], ciliary neurotrophic factor [9], and fibroblast growth factor-2 [10].

Most clinical studies on electrical stimulation of the eyeball use an electrical stimulation method with a corneal electrode. However, transcorneal electrical stimulation (TES), in which the electrodes are placed in direct contact with the corneal epithelium, may cause corneal epithelial damage. Therefore, we developed a transdermal electrical stimulation (TdES) device jointly with Mayo Co. Ltd. and performed a clinical trial to evaluate the safety and efficacy of transdermal electrical stimulation with skin electrodes in patients with RP. We confirmed that TdES did not cause any complications, and the visual acuity and mean deviation value of Humphrey field analyzer (HFA)10-2 significantly improved after TdES [11]. Based on these results, a larger, longer-term, multicenter, next-phase clinical trial is currently underway for RP patients [12].

Central retinal artery occlusion (CRAO) results in a severe loss of visual function by reducing or blocking the retinal blood flow including the macula, due to occlusion of the central retinal artery [13]. Hyperbaric oxygen [14], anticoagulation [15], retrobulbar tolazoline injection [16], sublingual isosorbide dinitrate [16], oral pentoxifylline [17], lowering intraocular pressure with topical ocular hypotensive agents, intravenous mannitol and acetazolamide, paracentesis, ocular massage [16], intravenous [18] or intra-arterial [19] thrombolysis have been reported as attempts to treat CRAO. However, strong evidence for effective treatments of CRAO after the acute phase has not yet been established.

Since one of the mechanisms of ocular electrical stimulation is improvement of the survival rate and functional activation of ganglion cells in the inner retina, electrical stimulation is considered effective for retinal artery occlusion.

Therefore, TES for retinal artery occlusion has been attempted, and improvements in the visual field and multilocal electroretinogram waveforms have been reported [20,21]. However, to the best of our knowledge, no studies have reported the use of TdES in CRAO patients.

Thus, the purpose of this exploratory clinical trial was to evaluate the visual function and safety of TdES in patients with CRAO.

## Materials and methods

This exploratory clinical trial was an investigator-led, prospective, non-randomized, open-label, uncontrolled multicenter trial conducted at Chiba University Hospital, Kobe University Hospital, and Nagoya City University Hospital in Japan. This was a 12 weeks trial consisting of six TdES treatments in patients with CRAO. TdES was applied every 2 weeks for 6 weeks, as in our previous trial on patients with RP [11]. This trial was registered in the UMIN Clinical Trials Registry (UMIN000036219) on March 15, 2019.

The diagnosis of CRAO was made based on a history of sudden and painless unilateral severe visual impairment and ophthalmoscopic findings, such as retinal edema (ischemic retinal whitening) and cherry red spot sign (due to underlying normal choroidal circulation) in the macula, as well as normal appearance of the optic disc.

Delay in retinal arterial filling, arteriovenous transit time, and normal choroidal vascular filling were confirmed by fluorescein angiography in the acute phase in all cases. Only patients with CRAO 6 months or longer after the onset were enrolled, as visual function may improve spontaneously more than 30 days after the onset [20].

The patients underwent TdES consisting of 10-ms biphasic pulses at 20 Hz for 30 min. Only the affected eye was simultaneously stimulated with 1.0-mA pulses in all patients by physicians. One electrode was placed on the lower eyelid lateral to the midline of the affected eye, and the other was placed at the center of the forehead. Electrical pulses were obtained from the same equipment used in a previous clinical trials jointly developed by Mayo Co., Ltd. (Aichi, Japan).

### Eligibility criteria

Eligible patients were those who met all of the following inclusion criteria and did not have any listed exclusion criteria.

### Inclusion criteria

1. Clinically diagnosed with CRAO and age ≥20 years and ≤80 years
2. CRAO patients who were determined to have fixed symptoms more than 6 months after the onset
3. Decimal visual acuity from hand motion to 0.7
4. Patients who provided signed consent with sufficient understanding after receiving an explanation of the responsibilities of participating in this trial
5. Regular hospital visits every 2 weeks for 12 weeks

### Exclusion criteria

1. History of allergy to mydriatic agents and eye surface anesthetics
2. Presence of vitreous macular traction syndrome, macular edema, epiretinal membrane, myopia with posterior staphyloma, diabetic retinopathy, conjunctival inflammation, ocular infection, severe dry eye, grade 3 or higher Emery-Little grade cataract, and posterior capsule opacification
3. History or complications of a malignant tumor. However, patients with a history of malignant tumor that has not relapsed for more than 5 years were not excluded
4. Diagnosis of dementia or mental disorder with ongoing treatment
5. Diabetes mellitus (HbA1c [NGSP]> 10.0%)
6. Hypertension (systolic ≥ 180 mmHg and/or diastolic ≥ 110 mmHg) that is difficult to control even with oral treatment
7. Any of the following on a screening blood sampling test:
  - Aspartate aminotransferase and alanine aminotransferase: more than three times the upper limit of the facility standard value
  - Serum creatinine: more than 1.5 times the upper limit of the facility standard value
8. Ongoing treatment with ethambutol hydrochloride and/or amiodarone hydrochloride
9. Ongoing pregnancy, breastfeeding, or possible or planned pregnancy during the trial period
10. Participation in other clinical trials
11. Under investigational responsibility (shared) judged by doctors to be inappropriate for participation in this trial

### Discontinuation criteria

1. When the subject offers to decline participation in the clinical trial or withdraws consent.
2. When the subject is found to be ineligible for the subject after enrollment in the clinical trial.
3. When it is difficult to continue the clinical trial due to exacerbation of complications.
4. When it is difficult to continue the clinical trial due to adverse events.
5. If pregnancy is found.
6. If the entire clinical trial is discontinued.
7. When other serious violations of the clinical trial protocol are found.
8. When the necessity of discontinuation is recognized at the discretion of the investigator.

The primary endpoint was the logarithm of the minimum angle of resolution (logMAR) visual acuity, and the secondary endpoints were changes in logMAR visual acuity, Early Treatment of Diabetic Retinopathy Study (ETDRS) visual acuity, mean deviation value of HFA 10–2, and score of HFA monocular Esterman test scores. The incidence of adverse events (AEs; type, frequency, and severity) was investigated for evaluation of safety.

Electrical stimulation was performed every 2 weeks for six sessions, and patients were assessed before TdES (baseline), 1 h after the end of treatment, and at 2 weeks after TdES. TdES treatment and examination schedule for this trial are shown in Table 1. The study protocol was approved by the institutional review boards of Chiba, Kobe, and Nagoya City University Hospital. Written informed consent was obtained from all patients before enrollment.

**Table 1:** Treatment and examination schedule.

	Screening	0 week	2 weeks	4 weeks	6 weeks	8 weeks	10 weeks	12 weeks	Drop out
	Visit 1–2	Visit 3	Visit 4	Visit 5	Visit 6	Visit 7	Visit 8	Visit 9	
IC	**○**								
TdES		**①**	**②**	**③**	**④**	**⑤**	**⑥**		
Blood pressure	**○**								
Blood sampling	**○**								
logMAR VA	**○**	**○**	**○**	**○**	**○**	**○**	**○**	**○**	**○**
ETDRS VA	**○**	**○**						**○**	**○**
HFA	**○**							**○**	**○**
Slit	**○**			**○**				**○**	**○**
IOP / fds	**○**			**○**				**○**	**○**
AEs	**○**								

**Abbreviations:** IC, informed consent; TdES, transdermal electrical stimulation; VA, visual acuity; HFA, Humphrey field analyzer; Slit, slit lamp examination; IOP, intraocular pressure; fds, fundus examination; AE, adverse event.

All patients underwent complete ophthalmic examination, including measurements of best-corrected visual acuity (BCVA) and intraocular pressure (IOP). In addition, slit lamp and indirect ophthalmoscopy examinations were performed. BCVA was measured monocularly using a Japanese standard Landolt ring chart.

Decimal visual acuities were converted to logMAR units for statistical analysis. We defined finger counting and hand motion as 1.8, light perception as 1.9, and the loss of light perception as 2.0 for logMAR BCVA.

BCVA was also assessed using the ETDRS chart (CSV-1000LanC VectorVision, Ohio, USA) at a distance of 2.5 m. The luminance for the tests was 85 cd/m2, which is the luminance recommended for vision testing by the USA National Academy of Sciences and adopted by the FDA as the required testing light level for clinical trials.

The mean deviation of retinal sensitivity and monocular Esterman test scores were determined using the Humphrey Visual Field Analyzer III (Model 850; Carl Zeiss Meditec, Inc., Dublin, CA, USA) with the Swedish Interactive Threshold Algorithm Standard 10–2 protocol and monocular Esterman test.

These methods were carried out in accordance with the relevant protocol.

### Statistical analysis

Demographic patient characteristics included age and sex by median, range (minimum to maximum), and frequencies. Clinical characteristics are presented as mean and standard deviation.

The primary and secondary endpoints were analyzed in the full analysis set, defined as all patients who underwent at least one TdES session and had at least one efficacy assessment. The results are presented as mean and mean difference from the baseline, 95% confidence intervals (CIs).

Mayo Co., Ltd. had no role in the study design, data collection, data analysis, data interpretation, or writing of the report. All authors had full access to all data in this trial, and the corresponding author was responsible for the decision to submit for publication.

## Results

**Fig 1:**
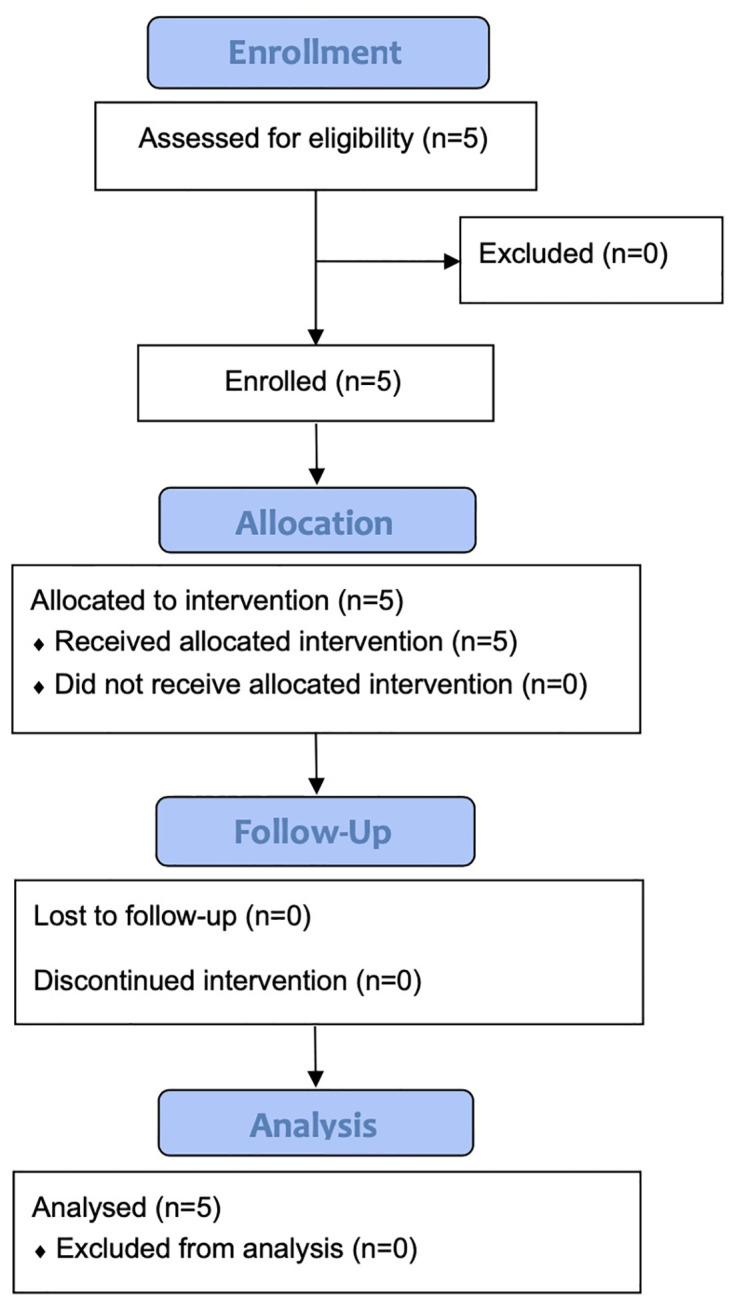
Study CONSORT flowchart.

Recruitment for this trial began in April, 2019 and ended in October, 2020, and patients were enrolled between June 25, 2019, and October 27, 2020. The end date for the last patient to complete the entire protocol was January 9, 2021. Five patients were screened, and no patients were excluded from the study. None of the cases were discontinued after enrollment (Fig 1). None of the cases showed CRAO with cilioretinal artery sparing. Finally, five eyes from five patients were included in the analyses.

The methods were performed in accordance with the relevant guidelines and regulations. The demographic and clinical characteristics of the patients at baseline and 12 weeks are shown in Table 2.

**Table 2:** Demographic and clinical characteristics at baseline and 12 weeks.

Parameters	Baseline	12 weeks
Number of patients / eyes	5 / 5	
Age (years)	66.6 (53 to 75)	
Male / Female	2 / 3	
logMAR VA	1.20 ± 0.41	1.18 ± 0.42
ETDRS VA (letters)	8.0 ± 17.33	5.0 ± 11.18
MD value of HFA10-2 (dB)	-31.25 ± 2.60	-30.19 ± 2.69
Score of HFA Esterman test	45.6 ± 37.34	44.8 ± 35.46

**Abbreviations:** VA, visual acuity; MD, mean deviation; HFA, Humphrey field analyzer.

Numerical values are the means ± standard deviation of the means.

The periods from the onset of CRAO to the initiation of TdES was 6–19 months (14 ± 5.8 months).

### Efficacy of the trial

**Fig 2:**
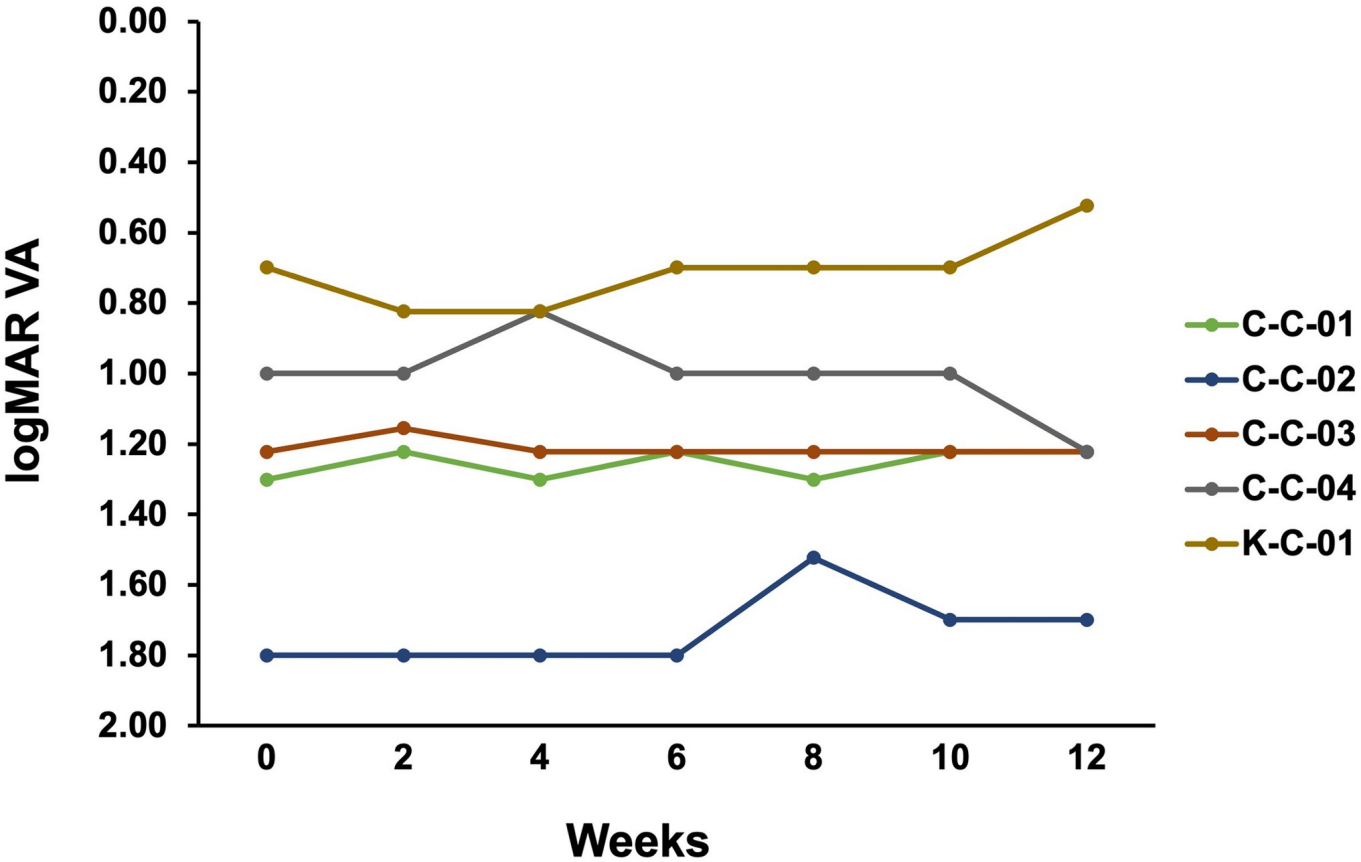
logMAR visual acuity for each case during the study period.

The logMAR for each patient is shown in Fig 2. The logMAR visual acuity at 12 weeks was the same as or better than the value at 0 weeks in four of five cases.

The change in logMAR visual acuity from 0 weeks was improved by 0.1 or more at 8, 10, and 12 weeks for patient C-C-02, at 4 weeks for patient C-C-04, and at 12 weeks for patient K-C-01. The rate of improvement in logMAR visual acuity from week 0 was 0.1 or more in 60% of cases (three of five cases). The change in logMAR visual acuity from 0 to 12 weeks varied greatly from case to case, and there was no consistent improvement in vision.

**Fig 3:**
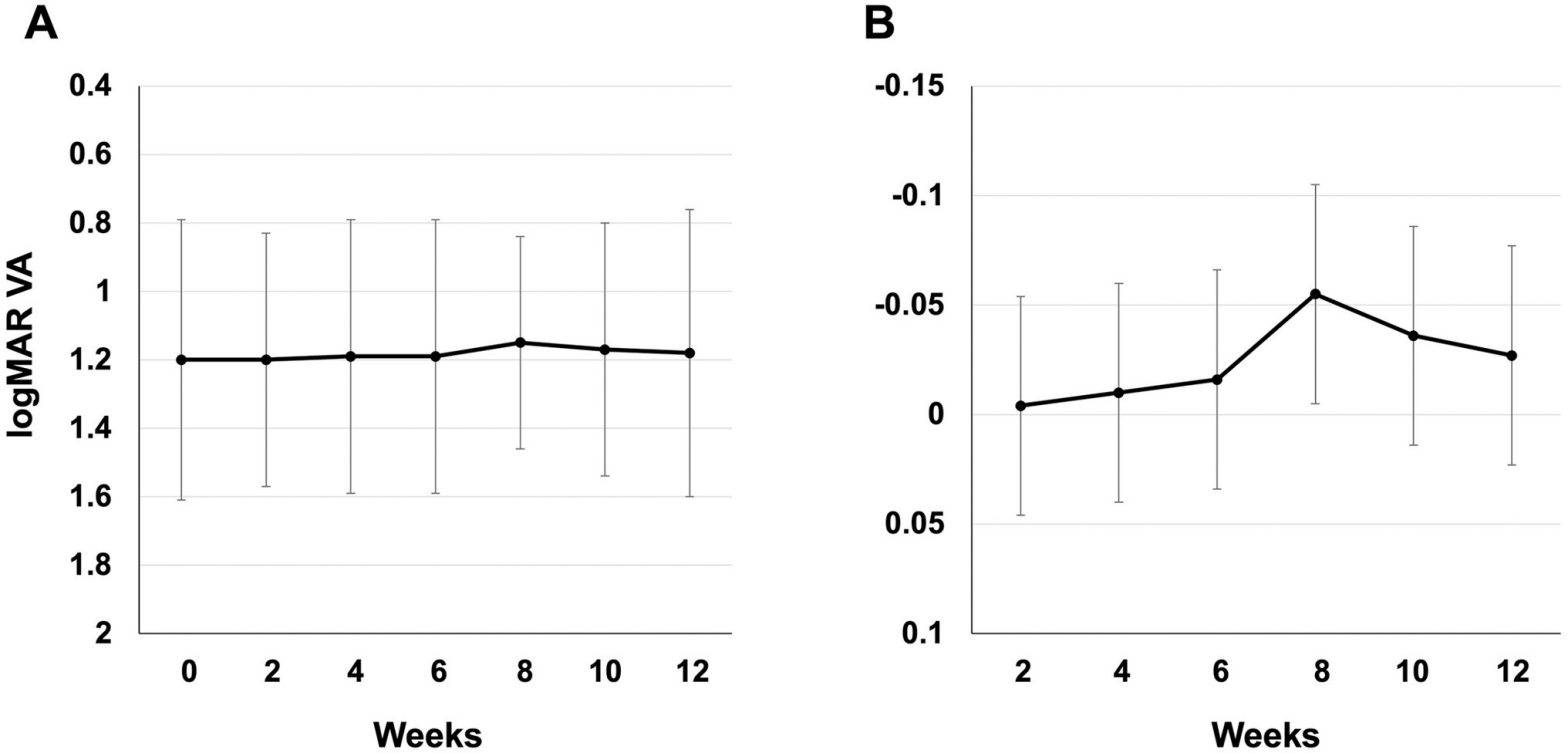
Mean and changes in logMAR visual acuity during the study period. (A) Mean logMAR visual acuity. Whiskers indicate standard deviation. (B) Changes in logMAR visual acuity. Whiskers indicate standard error.

The mean ± standard deviation and changes in logMAR visual acuity from the baseline values (0 weeks) throughout the study period (12 weeks) are shown in Fig 3. The mean logMAR visual acuity was 1.20 ± 0.41 at the baseline, 1.20 ± 0.37 at 2 weeks, 1.19 ± 0.40 at 4 weeks, 1.19 ± 0.41 at 6 weeks, 1.15 ± 0.31 at 8 weeks, 1.17 ± 0.37 at 10 weeks, and 1.18 ± 0.42 at 12 weeks (Fig 3A).

For comparison of logMAR visual acuity with week 0 in the mixed-effects model, the least mean square ± standard error was -0.004 ± 0.0449 (95% CI: [-0.098, 0.089]) at 2 weeks, -0.010 ± 0.0449 (95% CI: [-0.104, 0.083]) at 4 weeks, -0.016 ± 0.0449 (95% CI: [-0.110, 0.078]) at 6 weeks, -0.055 ± 0.0449 (95% CI: [-0.149, 0.038]) at 8 weeks, -0.036 ± 0.0449 (95% CI: [-0.130, 0.058) at 10 weeks, and -0.027 ± 0.0449 (95% CI: [-0.121, 0.067]) at 12 weeks (Fig 3B). Compared to week 0, the mean value of the change was higher than that at baseline, and no deterioration was observed in any week.

Table 2 shows the values of the logMAR visual acuity, ETDRS visual acuity, MD value of HFA 10–2, and HFA Esterman score at the baseline and final visit. The mean ETDRS visual acuity was 8.00 ± 17.33 (95%CI: [-13.52, 29.52]) letters at the baseline and 5.00 ± 6.16 (95%CI: [-10.65, 4.65]) letters at 12 weeks. None of the patients showed improvement in ETDRS visual acuity of 5 letters or more. The mean MD value of HFA 10–2 was -31.25 ± 2.60 (95%CI: [-34.48, -28.03]) dB at the baseline and -30.19 ± 4.60 (95%CI: [-35.90, -24.48]) dB at 12 weeks. The mean change ± standard deviation was 1.06 ± 2.69 (95%CI: [-2.27, 4.40]) dB. The mean HFA Esterman score was 45.60 ± 37.34 (95%CI: [-0.76, 91.96]) at the baseline and 44.80 ± 35.46 (95%CI: [0.77, 88.83]) at 12 weeks. The mean change ± standard deviation was -0.80 ± 7.79 (95%CI: [-10.47, 8.87]).

### Safety of the trial

The incidence of AEs (type, frequency, and severity) at 12 weeks was confirmed. No AEs occurred during the study. Similar to our previous results with TdES, abnormalities such as keratitis, dermatitis around the skin electrodes, inflammation of the anterior ocular segment, opacities of the optic media, abnormalities of the fundus and facial and trigeminal nerves, and nasal abnormalities were not observed. There were no significant differences in the intraocular pressure between the baseline and final visit values. The TdES was completed in all five cases, and there was no problem with tolerability to electrical stimulation.

## Discussion

To the best of our knowledge, this study is the first clinical trial to assess the efficacy and safety of transdermal electrical stimulation with a skin electrode in patients with CRAO.

Consequently, this study included CRAO patients with various baseline values. The change in logMAR vision from 0 to 12 weeks in each case showed a large difference among the cases, and no constant tendency to improve vision was observed. Similar results were obtained for ETDRS visual acuity, MD, and Esterman test scores of the HFA.

An increase in the level of IGF-1 [7], ciliary neurotrophic factor, brain-derived neurotrophic factor [9], and fibroblast growth factor-2 [8], upregulation of Bcl-2 expression and downregulation of Bax expression [9], inhibition of the NF-kB signaling pathway, and suppression of microglial activation [21] have been proposed as mechanisms for improving visual function by ocular electrical stimulation. Retinal ganglion cells (RGC) in patients with CRAO was severely and extensively impaired due to CRAO compared to RP, non-arteritic ischemic optic neuropathy, Leber’s hereditary optic neuropathy, and glaucoma, for which TES has been reported to improve visual function.

Phosphenes are visual perceptions induced by stimuli other than light. Electrically evoked phosphene thresholds have been used as indicators of electrical stimulation of RGC and have been used, for example, in the evaluation of implanted artificial retinas. Naycheva et al. investigated electrical phosphene thresholds in healthy subjects and patients with retinal diseases and glaucoma [22]. They reported that the electrical phosphene thresholds at all frequencies were lowest in healthy subjects and highest in those with retinal artery occlusion. Electrical stimulation therapy is assumed to be effective when the patient’s retinal ganglion cell is alive but dysfunctional or when progenitor cell plasticity remains [23]. Therefore, CRAO, which has a high phosphene threshold and causes severe and widespread death of RGC, may be a disease in which the effect of electrical stimulation is less likely to be obtained than in other diseases.

Heyreh et al. reported that CRAO improved spontaneously in two of 21 eyes (10%) even 1 month after onset [20]. Since we tried to eliminate the effects of natural remission and verify only the effects of electrical stimulation, the study protocol was set up to include only cases 6 months after onset in this study. Further studies are needed to determine the consequences of introducing treatment early after CRAO onset.

There are very few reports of electrical stimulation therapy for CRAO. Inomata et al. performed TES therapy (20 Hz, 30 minutes, up to 1100 μA, once a month for 3 months) in two patients with CRAO (15 and 33 months, respectively), and one with branch RAO (26 months) and reported that the BCVA improved by more than 0.2 logMAR units in the two CRAO cases and the visual fields of Goldmann perimetry were improved in all the three cases [24]. Although the number and frequency of treatments were higher in our study than in Inomata’s report, there were no cases of improvement in 0.2 logMAR unit or more, and the Esterman test, which is an alternative to GP, did not show any significant improvement in our study. It was unclear why our study did not show the same visual function improvement effect as their results. The different types of electrodes, different baselines, and smaller sample sizes in both the studies may be related to the different results. Neycheva et al. reported that 12 patients with CRAO and one patient with branch RAO were treated with TES at 0 mA (sham, n = 3), 66% (n = 5), or 150% (n = 5) of the patient’s individual electrical phosphene threshold at 20 Hz for 30 min once a week for 6 consecutive weeks [25]. Their results showed that statistically significant improvements were found only for specific a-wave slopes, and BCVA, HFA, GP, and other electroretinogram (ERG) parameters showed no significant changes. We did not evaluate ERG, but the other results were similar. They speculated that the reason they did not reach statistical significance was the very small sample size and the short study period of six stimulation sessions. This consideration was consistent with our conditions.

Decimal visual acuities were converted to logMAR units for statistical analyses in this study because the decimal chart prevails in Asia in clinical practice.

However, previous studies have demonstrated that decimal charts have several disadvantages [26]. A decrease in measurement precision and reproducibility across different charts is induced because decimal charts have not been designed to contain optotypes that have an equivalent logMAR value, irrespective of the acuity line. The mean ETDRS visual acuity was reduced even though the mean logMAR visual acuity was maintained. As for ETDRS vision, there were three patients who had 0 letters at both baseline and 12 weeks, one patient who had 1 and 0 letters, and one patient who had 39 and 25 letters. Ophthalmic findings in patients with ETDRS vision reduced from 39 to 25 characters did not worsen during the study period; therefore, it was unclear why these results were obtained. Recently, Mataftsi et al. showed disagreement between VA scores with decimal charts and those on ETDRS charts, especially in patients with poorer acuity [27]. These phenomena may be one of the reasons for the difference in the logMAR and ETDRS visual acuity results shown in this study. Methods for evaluating visual function in patients with low vision, such as CRAO patients, are not well defined, and a more reproducible method is expected to be established.

Regarding safety evaluation, electrical stimulation therapy was completed according to the protocol for all five patients. No patient dropped out due to irritation or pain caused by electrical stimulation, and there was no problem with tolerability to electrical stimulation. No AEs related to this therapy were observed during the study. Thus, it can be concluded that TdES was safe under the stimulation protocol implemented in this study.

The limitations of this study include the small number of cases, lack of control groups, and short treatment and observation periods. Larger long-term trials with control groups are required. Due to these limitations, we cannot conclude about the efficacy of TdES for CRAO in this study. Furthermore, as mentioned above, the effect of CRAO in the early post-onset period, which is expected to be more effective, has not been verified; the phosphene thresholds and ERG have not been measured. Evaluating these parameters may provide insight into the relationship between the residual retinal function and the effectiveness of electrical stimulation therapy.

In conclusion, the effects of TdES therapy on visual acuity and visual field in CRAO patients were unclear in this exploratory trial; however, no serious AEs occurred, and the treatment was tolerated. The logMAR visual acuity was slightly improved and was maintained in two patients in this study, and the safety of TdES treatment was confirmed in both this study and our previous study in patients with RP (11). Since CRAO has no established treatment method, further research into the effects of TdES treatment in CRAO patients may be beneficial.

## Supporting information

S1 Checklist CONSORT 2010 checklist of information to include when reporting a randomised trial*. (DOC)

S1 File (DOCX)

S2 File (DOCX)

S1 Dataset (XLSX)
